# Lanthanum-modified shale powder/alginate composite hydrogel beads for fluoride-contaminated groundwater treatment

**DOI:** 10.3389/fchem.2026.1962172

**Published:** 2026-09-23

**Authors:** Sitong Liu, Shaoxiong Li, Xu Bi, Guangying Feng, Zhao Wang, Qiusheng Yuan, Yue Xu, Ningyi Li

**Affiliations:** 1 Academic Affairs Office, Kunming Railway Vocational and Technical College, Kunming, Yunnan, China; 2 Faculty of Environmental Science and Engineering, Kunming University of Science and Technology, Kunming, Yunnan, China

**Keywords:** alginates, composite hydrogel beads, fluoride, lanthanum modification, shale powder

## Abstract

Excessive fluoride (F^−^) in groundwater poses a serious threat to human health, necessitating the development of efficient defluorination materials. In this study, a novel composite hydrogel bead (La-SP/SA) was fabricated by incorporating shale powder (SP) into sodium alginate (SA) matrix followed by crosslinking with lanthanum chloride. The synthesis conditions were determined as SP dosage 4.0%, La^3+^ concentration 0.2 mol L^−1^, and crosslinking time 12 h. Under adsorbent dosage of 0.7 g L^−1^, initial F^−^ concentration of 9.0 mg L^−1^, and pH of 5.0, La-SP/SA achieved an excellent removal efficiency of 94.33% (12.18 mg g^−1^), with a residual F^−^ concentration meeting both the WHO guideline and Chinese standard. Kinetic analysis revealed that the adsorption process followed the pseudo-second-order model, characteristic of chemisorption-dominated systems. The composite hydrogel beads exhibited excellent selectivity toward F^−^ in the presence of coexisting anions (Cl^−^, SO_4_^2−^, HCO_3_^−^, and NO_3_^−^) and maintained over 98.01% of initial adsorption capacity over 5 days across pH 4.0–9.0. Mechanistic investigations using SEM-EDS, FTIR, XRD, and XPS demonstrated that F^−^ removal occurred via a synergistic mechanism involving electrostatic interaction, ion exchange, inner-sphere complexation, chemical precipitation, and chemisorption. This work provides a promising strategy for fluoride-contaminated groundwater remediation while enabling the resource utilization of shale waste.

## 1 Introduction

Groundwater with high fluoride (F^−^) concentrations poses a significant risk to human health (Huyen et al., 2023). Chronic excessive intake of F^−^ (1.5 mg L^−1^) can trigger dental and skeletal fluorosis and impair kidney and liver function (Wang and Wu, 2025). It has also been linked to an increased incidence of cancer, thyroid dysfunction, and Alzheimer’s disease (Yu M. et al., 2025). Emerging evidence further indicates that excessive F^−^ exposure affects the PTH hormone secretion and calcium homeostasis (Thakur and Patial, 2026). At the cellular level, the F^−^ is known to trigger oxidative stress and apoptosis through the generation of reactive oxygen species and disruption of mitochondrial function (Wang and Wu, 2025). Additionally, F^−^ contamination exerts toxic effects on the growth and reproduction of both animals and plants (Lacson et al., 2021). In animals, the F^−^ exposure has been associated with reduced fertility, impaired embryonic development, and damage to reproductive organs. In plants, the F^−^ toxicity manifests as inhibited seed germination, reduced photosynthetic activity, and decreased biomass production. It is noteworthy that F^−^ at concentrations between 0.5 and 1.0 mg L^−1^ is beneficial for dental health by preventing tooth decay (Jian et al., 2025). However, levels exceeding this range can induce adverse health outcomes (Shen et al., 2022). Recognizing these health risks, the World Health Organization (WHO) has established a guideline value for F^−^ concentration in drinking water as 1.5 mg L^−1^, while China has implemented a stricter standard, limiting F^−^ levels to 1.0 mg L^−1^. Therefore, effective management and reduction of F^−^ concentrations in groundwater are essential to ensure the safety of drinking water.

Among various defluorination techniques, adsorption is widely considered one of the most promising approaches due to its operational simplicity, cost-effectiveness, and stability (Liu S. et al., 2025; Zeng et al., 2023). The effectiveness of this process primarily stems from the high specific surface area, hierarchical pore structure, and abundant surface functional groups of adsorbents, which immobilize F^−^ via physical adsorption or chemical complexation, thereby enabling its efficient removal from aqueous solutions (Zeng et al., 2023). In recent years, hydrogel-based adsorbents have attracted growing attention in the water treatment field owing to their unique three-dimensional network structures and exceptional adsorption capabilities (He et al., 2020). Sodium alginate (SA), a natural polysaccharide, is particularly attractive due to its low cost, biocompatibility, and biodegradability (Njaramba et al., 2022). Hydrogel beads fabricated from SA have been demonstrated considerable potential for the adsorption of various pollutants (Dong et al., 2025). Whereas, pure sodium alginate hydrogels exhibit limited adsorption capacity and low selectivity toward F^−^ (Chang et al., 2024), which require modification to enhance their performance.

In recent years, the combination of solid waste materials with biopolymer matrices has attracted increasing attention for water treatment applications. For instance Xu et al. (2026) successfully fabricated lanthanum-calcium modified coal gangue/alginate composite beads for F^−^ removal, demonstrating the feasibility of integrating mining by-products into hydrogel systems. Shale powder (SP), a solid waste generated from shale gas extraction and mining operations, is primarily composed of clay minerals (e.g., kaolinite and illite), fine-grained quartz, and feldspar. Its layered silicate structure and inherent hierarchical porosity provide high specific surface area and rich silanol groups (Xu et al., 2025), which may promote stronger interactions with alginate and more favourable binding sites for F^−^. Previous studies have proved that incorporating inorganic or organic fillers into hydrogel matrices can trigger synergistic improvements in adsorption capacity, mechanical strength, and material durability (Dong et al., 2025). The integration of shale as a functional component into hydrogel systems not only facilitates the resource utilization of solid waste but is also anticipated to enhance the overall adsorption performance of the composite material. Lanthanum (La), a rare earth element, has garnered significant attention as a modifier for defluorination materials owing to its strong affinity for anions (Zhang et al., 2025). This high affinity is attributable to the unique physicochemical properties of La, which possess a large ionic radius and high charge density. These characteristics promote the generation of a strong localized electrostatic field, thereby effectively sequestering F^−^ from aqueous solutions (Liu et al., 2024). Embedding La within a SP/SA composite system leverages the exceptional fluoride-capturing capability of La, which is expected to further enhance the adsorption capacity and selectivity of the composite system. Nevertheless, no study to date has integrated SA, SP, and La into a single composite system for F^−^ removal, and their synergistic effects remain unexplored.

This study aims to fabricate lanthanum-modified shale powder/alginate composite adsorbents (La-SP/SA) and optimize their preparation (SP dosage, La^3+^ content, and crosslinking time) and operation (adsorbent dosage, initial F^−^ content, and initial pH value) parameters. The adsorption behavior was determined using kinetic models. Competitive adsorption experiments were conducted to assess the selectivity of La-SP/SA toward F^−^. Ultimately, the defluorination mechanisms were comprehensively investigated through a suite of characterization techniques.

## 2 Materials and methods

### 2.1 Chemicals and materials

SP, obtained from a mineral processing factory in Hebei, China, was primarily composed of quartz and calcite. The as-received material was dried at 105 °C for 24 h to remove moisture and then passed through a 300-mesh sieve to ensure particle size uniformity. The sieved powder was stored in a desiccator prior to use. All chemical reagents (analytical grade) were purchased from Macklin Chemical Reagent Co., Shanghai, China.

### 2.2 Preparation of composite hydrogel beads

The La-SP/SA preparation process is shown in Scheme 1. SA was dissolved in deionized water (2.0%, w/v) under continuous magnetic stirring, followed by ultrasonication to achieve complete dissolution. A predetermined amount of SP (0.0%, 1.0%, 2.0%, 4.0%, and 6.0%, expressed as weight percentage (w/w) relative to the mass of deionized water used in the preparation) was then introduced into the solution, and further ultrasonication was applied to obtain a homogeneous mixture. This mixture was subsequently injected dropwise via a syringe into an aqueous solution of lanthanum chloride (0.1, 0.2, 0.5, 1.0, and 2.0 mol L^−1^), where it underwent crosslinking (8, 12, 24, 36, and 48 h) to form composite hydrogel beads. The beads were thoroughly washed with deionized water until a neutral pH was attained, then freeze-dried and stored in sealed containers for further use.

**SCHEME 1:**
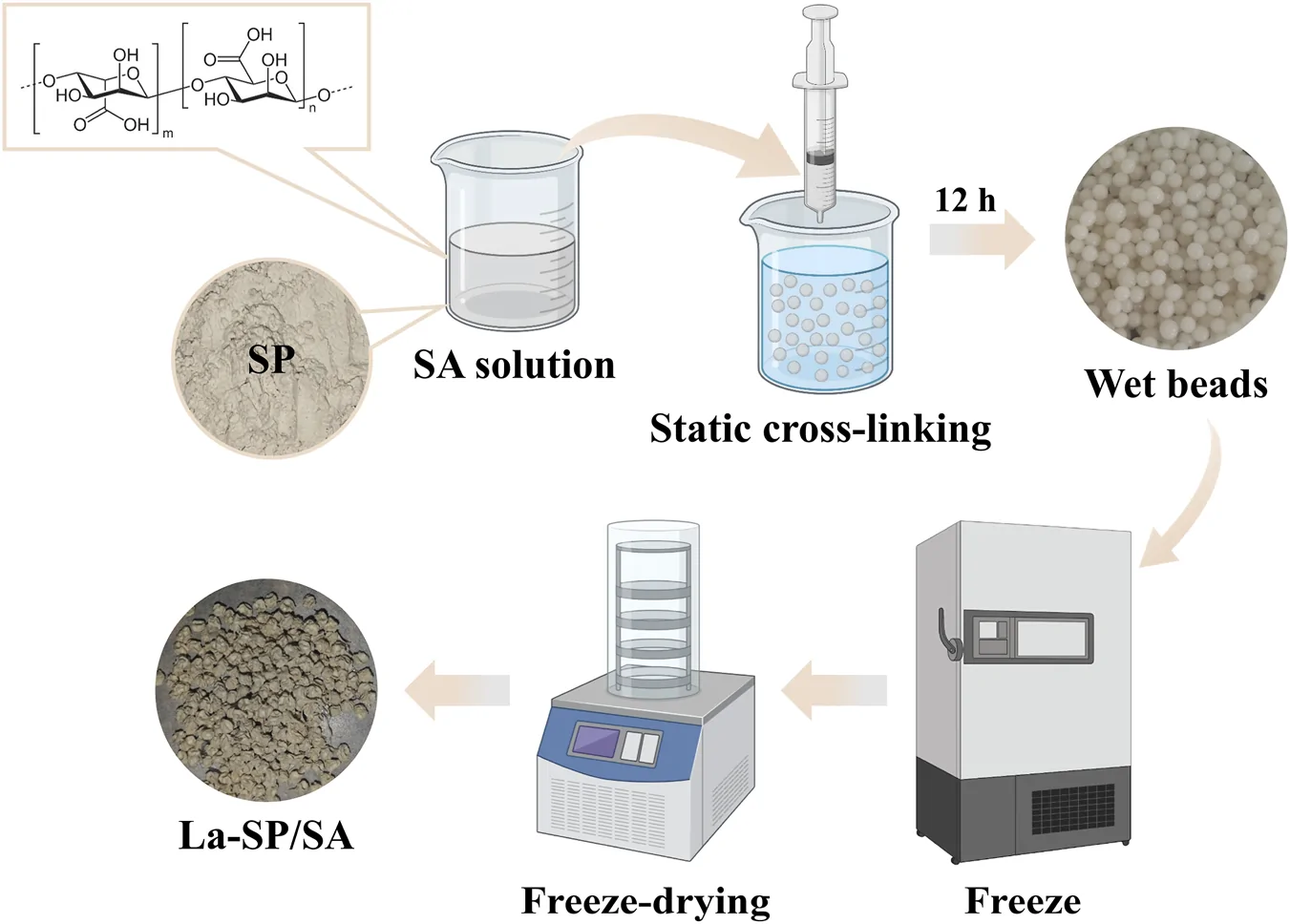
Synthesis of La-SP/SA composite hydrogel beads.

### 2.3 Adsorption experiments and kinetics

All batch adsorption experiments were conducted in 50 mL conical flasks containing 50 mL of F^−^ solution with a predetermined amount of La-SP/SA adsorbent. Unless otherwise specified, the conditions for all adsorption experiments were set as pH 7.0, initial F^−^ concentration of 5.0 mg L^−1^, and adsorbent dosage of 0.5 g L^−1^. The conical flasks were shaken at 180 rpm and room temperature (approximately 25 °C). At designated time intervals, samples were collected and filtered through 0.45 μm membrane filters. To ensure constant ionic strength and minimize electrode variability, total ionic strength adjustment buffer was added prior to measurement. The adsorption capacity (q_t_, mg g^−1^) and the removal efficiency R, %) were determined using (Equations 1 and 2), respectively.

$$q=\frac{\left(C_{0}-C_{e}\right)\times V}{M}$$ (1)

$$R=\frac{C_{0}-C_{e}}{C_{0}}\times 100\%$$ (2)

where C_0_ and C_e_ (mg L^−1^) are the initial and equilibrium F^−^ concentrations, respectively. V (L) and m (g) are the solution volume and adsorbent mass, respectively.

To elucidate the adsorption kinetics of F^−^ onto La-SP/SA, three classical kinetic models, including pseudo-first order (Equation 3), pseudo-second order (Equation 4), and intra-particle diffusion (Equation 5), were employed.

$$\ln\left(q_{e}-q_{t}\right)=\ln\left(q_{e}\right)-k_{1}t$$ (3)

$$\frac{t}{q_{t}}=\frac{1}{{\left(k_{2}q_{e}\right)}^{2}}+\frac{t}{q_{e}}$$ (4)

$$q_{t}=k_{i}t^{0.5}+C_{i}$$ (5)

where q_e_ denotes the equilibrium adsorption capacity (mg g^−1^). k_1_, k_2_, and k_i_ represent the rate constants of various kinetics, respectively. C_i_ is a constant related to the boundary layer thickness. All kinetic experiments were conducted in triplicate and the mean values were used for model fitting.

### 2.4 Characterization and analytical methods

The F^−^ concentration was measured using a fluoride-selective electrode (PXSJ-216F, INESA, China), and the Cl^−^ content was determined by silver nitrate titration. An inductively coupled plasma optical emission spectrometer (ICP-OES, 8900, Agilent, USA) was employed to determine the concentrations of La^3+^ and Ca^2+^. The microstructure of the composite hydrogel beads was examined using scanning electron microscope equipped with an energy dispersive spectrometer (SEM-EDS, OXFORD Ultim Max 100, Oxford Instruments, United Kingdom). The surface functional groups and crystalline structure were characterized by Fourier transform infrared spectroscopy (FTIR, Frontier, PerkinElmer, United States of America) and X-ray diffraction (XRD, D8, Bruker AXS, Germany). X-ray photoelectron spectroscopy (XPS, Nexsa, Thermo Scientific, USA) was employed to compare the binding energies of relevant elements before and after F^−^ adsorption. Additionally, the point of zero charge (pH_PZC_) of La-SP/SA was determined via the pH drift method. All experiments were conducted in triplicate, and data were recorded as mean values. Data processing and kinetic model fitting were performed using Origin 2026, while XPS spectra and XRD patterns were analyzed using Avantage 6.9 and MDI Jade 6.0, respectively.

## 3 Results and discussion

### 3.1 Optimization of synthesis parameters

#### 3.1.1 Shale powder dosage

The impact of SP dosage (0.0%, 1.0%, 2.0%, 4.0%, and 6.0%) on F^−^ removal was investigated. According to Figure 1a, the F^−^ removal efficiency increased from 87.83% (9.00 mg g^−1^) to 90.03% (9.23 mg g^−1^) as the SP dosage rose from 0.0% to 4.0%, indicating that the incorporation of SP enhanced the adsorption capacity of the hydrogel beads toward F^−^, which can be attributed to the strong affinity between SP and F^−^ (Zhao et al., 2025). However, a further increase in SP dosage caused a slight reduction in removal efficiency, which may be owing to factors such as particle aggregation (Behera and Sahu, 2023). Hence, the SP dosage was determined as 4.0% in subsequent experiments.

**FIGURE 1:**
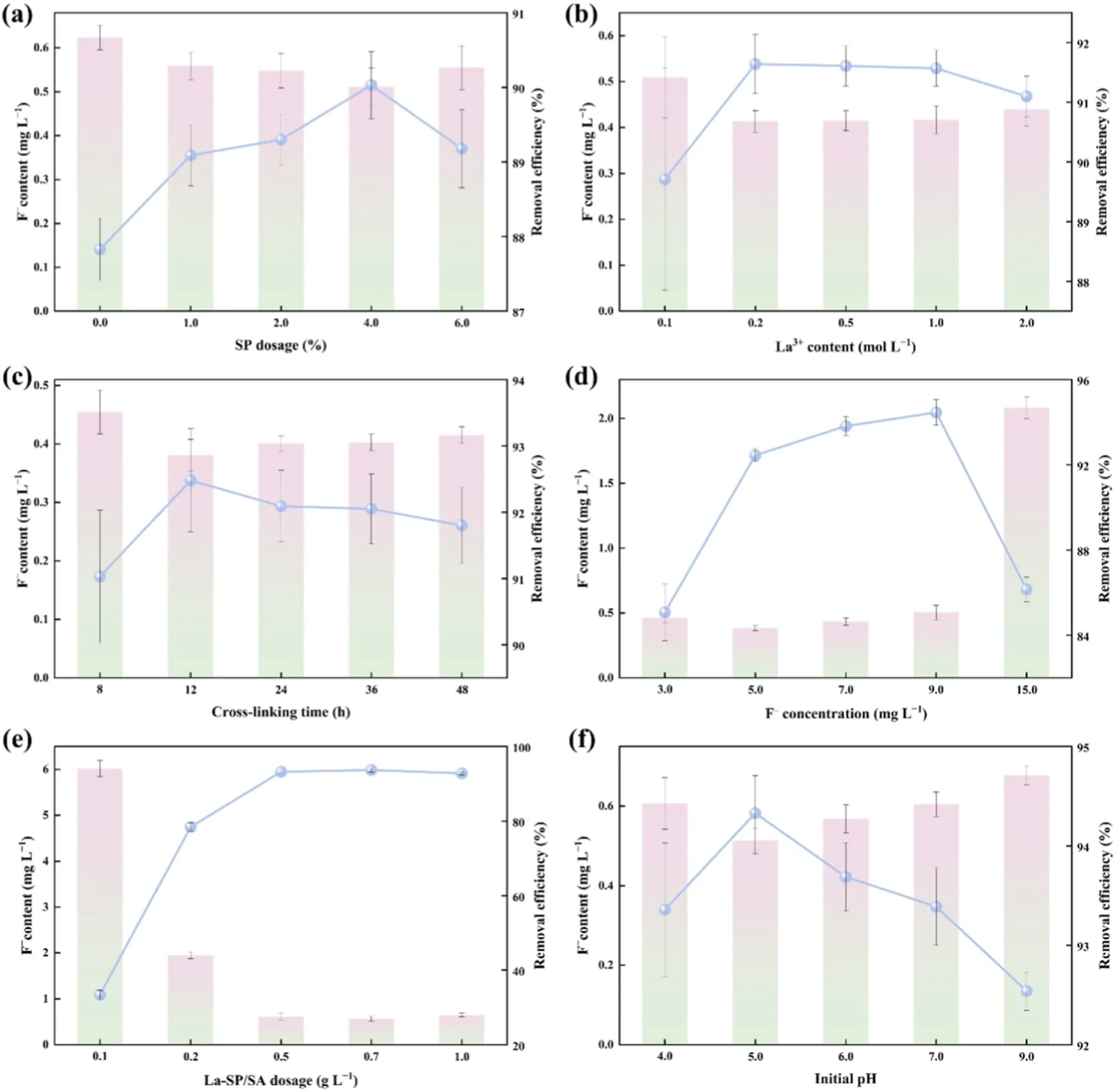
Optimization of **(a–c)** synthesis parameters (SP dosage, La^3+^ content, and crosslinking time) and **(d–f)** operation parameters (F^−^ concentration, La-SP/SA dosage, and initial pH).

#### 3.1.2 Lanthanum content

The influence of La^3+^ concentration (0.1, 0.2, 0.5, 1.0, and 2.0 mol L^-1^) on F^−^ adsorption was examined. With the increasing La^3+^ concentration from 0.1 to 0.2 mol L^−1^, the F^−^ removal efficiency rose from 89.71% (8.88 mg g^−1^) to 91.64% (9.07 mg g^−1^). However, the La^3+^ concentration beyond 0.2 mol L^−1^ did not cause a significant enhancement in adsorption performance (Figure 1b). This trend aligned with previous research, where the F^−^ adsorption onto lanthanum-modified spherical magnesium oxide tended to stabilize as the La impregnation ratio varied from 10% to 20% (Zhang et al., 2022). Within the lower concentration range, the number of active adsorption sites increased with growing La^3+^ concentration. Once the concentration exceeded a threshold (0.2 mol L^−1^), the adsorption sites became saturated, triggering a plateau in adsorption efficiency (Jeyaseelan et al., 2023). Considering both economic costs and adsorption performance, 0.2 mol L^−1^ was selected as the optimal La^3+^ concentration within the tested range.

#### 3.1.3 Crosslinking time

Figure 1c illustrated the F^−^ adsorption performance at various crosslinking time (8, 12, 24, 36, and 48 h). Experimental results indicated that extending the crosslinking time from 8 to 12 h increased F^−^ adsorption efficiency from 91.03% (9.22 mg g^−1^) to 92.48% (9.37 mg g^−1^). Further prolongation of the crosslinking time caused a slight decline in adsorption efficiency. The longer crosslinking facilitated the formation of additional adsorption sites and enhanced the structural stability of the material, thereby improving adsorption performance (Ratthika et al., 2025). However, excessive crosslinking may cause over-densification of the network structure or blockage of active sites (Rosendo-González et al., 2024), triggering a reduction in adsorption capacity. Therefore, the crosslinking time was identified as 12 h in subsequent experiments.

### 3.2 Optimization of operational parameters

#### 3.2.1 F^−^ concentration

The effect of initial F^−^ concentration (3.0, 5.0, 7.0, 9.0, and 15.0 mg L^−1^) on the adsorption performance was investigated (Figure 1d). The F^−^ removal efficiency increased from 85.08% (5.28 mg g^−1^) to 94.46% (17.21 mg g^−1^) with rising F^−^ concentration from 3.0 to 9.0 mg L^−1^. This was consistent with previous observations (Dar and Kurella, 2024), which can be attributed to the enhanced driving force for mass transfer, promoting the utilization of active sites on La-SP/SA (Azzeddine et al., 2024). As the initial F^−^ concentration further increased to 15.0 mg L^−1^, the F^−^ removal efficiency declined to 86.15% (25.91 mg g^−1^), which was owing to the saturation of these available binding sites (Hussain et al., 2025). Among the tested concentrations, the highest removal efficiency was achieved at an initial F^−^ concentration of 9.0 mg L^−1^, which also yielded a residual F^−^ concentration meeting the WHO guideline. Therefore, 9.0 mg L^−1^ was selected as the initial F^−^ concentration for subsequent experiments.

#### 3.2.2 La-SP/SA dosage

Adsorbent dosages ranging from 0.1 to 1.0 g L^−1^ were added into the F^−^ solution to assess the impact of adsorbent dosage. As illustrated in Figure 1e, the F^−^ removal efficiency rose from 33.44% (30.24 mg g^−1^) to 93.69% (12.10 mg g^−1^) as the adsorbent dosage increased from 0.1 to 0.7 g L^−1^. This can be attributed to the incremental availability of adsorption sites at higher adsorbent dosage (Li B. et al., 2025). However, further increase in the adsorbent dosage beyond 0.7 g L^−1^ did not cause a significant improvement in F^−^ removal by La-SP/SA. Azzeddine et al. (2024) also observed a similar trend, and attributed it to the steric effects induced by excessive adsorbent, which render the remaining active sites inaccessible. Notably, La-SP/SA achieved a residual F^−^ content meeting the WHO standard at the dosage of 0.7 g L^−1^, while maintaining an experimental adsorption capacity of 12.10 mg g ^−1^. Therefore, 0.7 g L^−1^ was identified as the optimal La-SP/SA dosage within the tested range.

#### 3.2.3 Initial pH

The pH value of the solution played a critical role in the adsorption process, as it influenced the surface charge of the adsorbent (Yan and Li, 2021). The effect of pH was evaluated across a range of 4.0–9.0. As illustrated in Figure 1f, the optimal maximum F^−^ removal efficiency (94.33% and 12.18 mg g^−1^) was recorded at pH 5.0, with lower efficiencies under more acidic or alkaline conditions. The pH_PZC_ of La-SP/SA was determined to be 6.18 (Figure 2). Below this pH, the surface was positively charged due to protonation of functional groups, which facilitated electrostatic attraction of F^−^ (Bi et al., 2026). However, at lower pH (<5.0), the reaction between H^+^ and F^−^ to form hydrogen fluoride hindered the binding of F^−^ species to the active sites on the adsorbent surface, reducing the adsorption capacity (Azzeddine et al., 2024). Above the pH_PZC_, the surface became negatively charged, triggering electrostatic repulsion between the adsorbent and F^−^, along with competitive adsorption between OH^−^ and F^−^ (Oke et al., 2023). Notably, the relatively modest decline from pH 5.0 to 9.0 indicated that non-electrostatic mechanisms dominated the adsorption process at near-neutral and alkaline conditions, effectively compensating for the loss of electrostatic attraction. Notably, La-SP/SA thus maintained relatively stable adsorption performance across a wide pH range (5.0–9.0), indicating its potential applicability.

**FIGURE 2:**
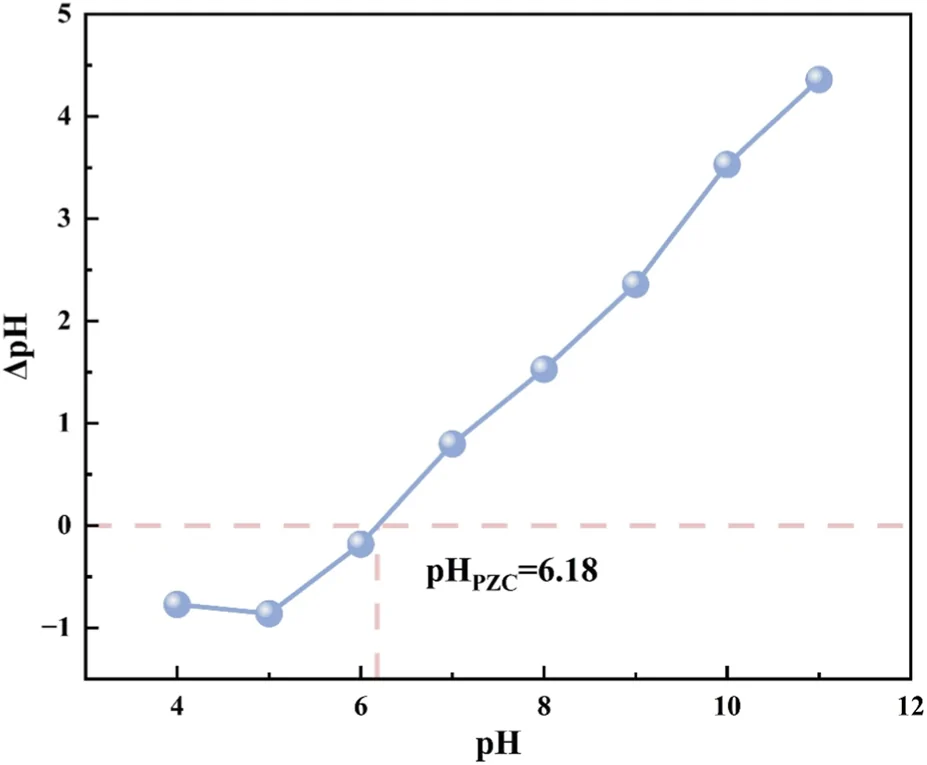
Point of zero charge (pH_pzc_) of La-SP/SA.

### 3.3 Adsorption kinetics and rate-controlling mechanism

Three kinetic models (pseudo-first-order, pseudo-second-order, and intra-particle diffusion) were applied to identify the rate-controlling step. The fitting parameters were summarized in Supplementary Tables S1-S6. As depicted in Figures 3, 4, the pseudo-second-order model exhibited excellent linear relationships under all tested conditions, with correlation coefficients (R^2^) consistently exceeding 0.97. Furthermore, the calculated equilibrium adsorption capacities (q_e_, _exp_) derived from this model closely matched the experimental values (q_e_, _cal_). These results indicated that the adsorption kinetics were best described by the pseudo-second-order model, and chemisorption was the predominant rate-controlling step (Aljohani, 2025). Further evidence was presented in Sections 3.5 and 3.6.

**FIGURE 3:**
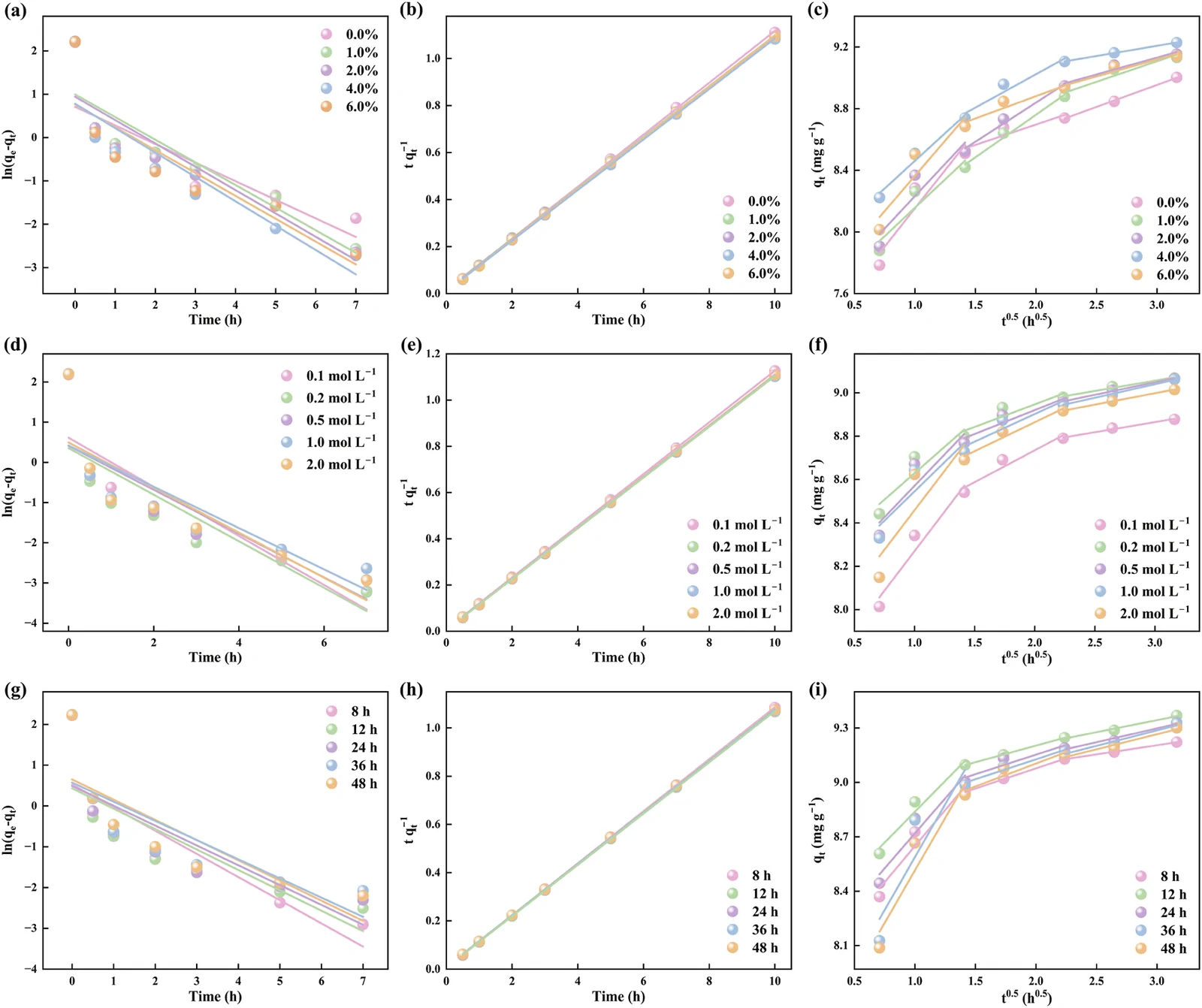
Fitting results of pseudo-first order, pseudo-second order, and intra-particle diffusion models under various **(a–c)** SP dosage, **(d–f)** La^3+^ content, and **(g–i)** crosslinking time.

**FIGURE 4:**
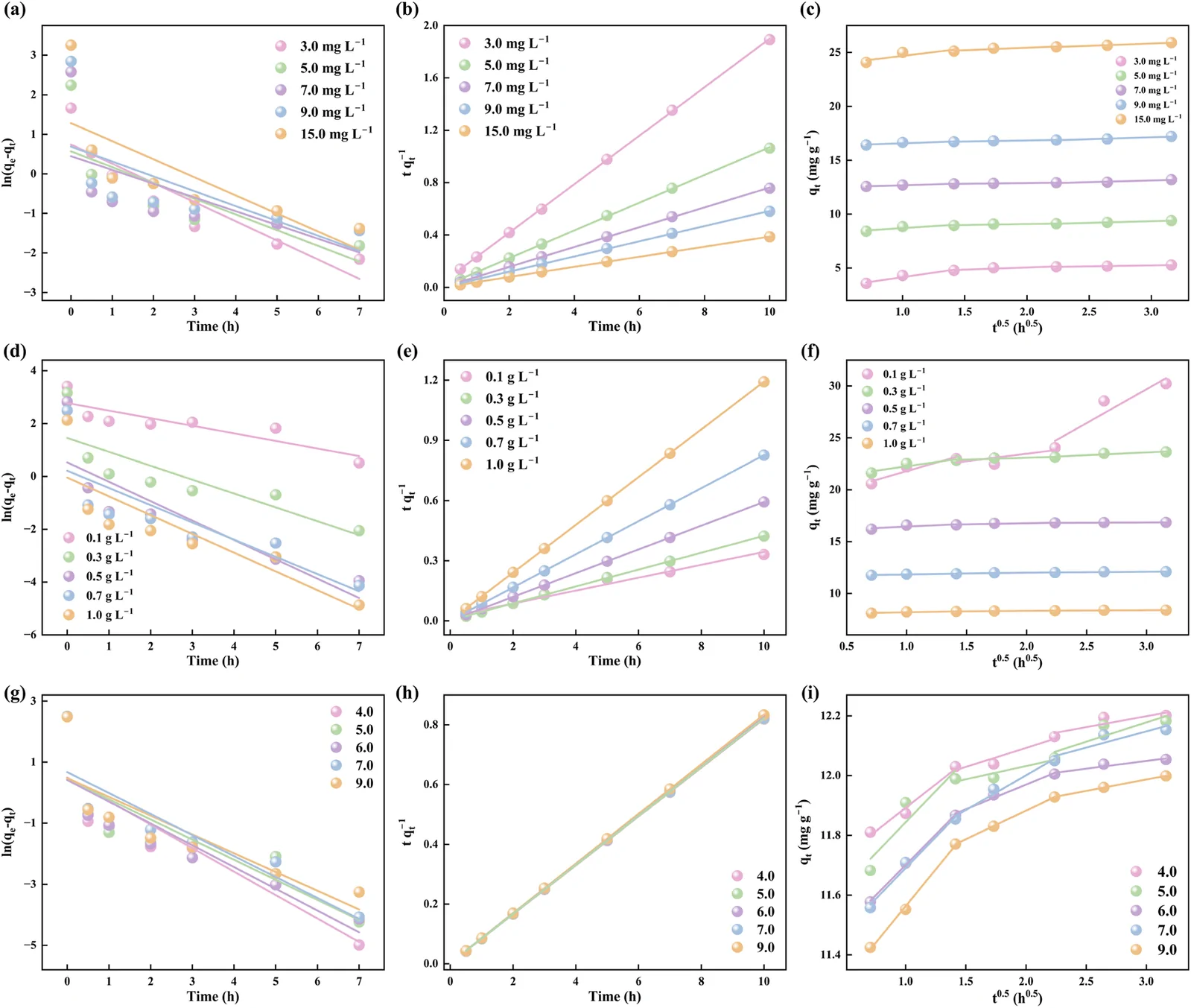
Fitting results of pseudo-first order, pseudo-second order, and intra-particle diffusion models under various **(a–c)** F^−^ concentration, **(d–f)** La-SP/SA dosage, and **(g–i)** initial pH.

The intra-particle diffusion model exhibited a multi-linear trend (Figures 3, 4), which could be broadly divided into three stages, a phenomenon commonly observed in porous adsorbents (Wang et al., 2021). In most cases, the initial linear segment exhibited a steeper slope, indicating a higher adsorption rate primarily governed by external diffusion (Foroutan et al., 2025). Subsequently, the linear region displayed a reducing slope, suggesting that the adsorption process transitioned to being controlled by pore diffusion. The final linear segment corresponded to the equilibrium stage, where intra-particle diffusion significantly slowed (Yusuff et al., 2025). Non-zero intercepts in all fitted curves indicated that intra-particle diffusion was not the sole rate-controlling step for F^−^ adsorption on La-SP/SA (Yu S.-H. et al., 2025).

### 3.4 Application feasibility

#### 3.4.1 Adsorption stability of La-SP/SA

The adsorption stability of La-SP/SA was investigated through a five-day leaching experiment across a pH range from 4.0 to 9.0. According to Figure 5, the composite beads exhibited excellent F^−^ retention capacity across all tested pH values, retaining over 98.01% of the maximum adsorption capacity. This indicated their robust performance stability under conditions relevant to natural water (Linhardt, 2026). In contrast to unmodified alginate beads, which are known to be susceptible to chemical degradation (He et al., 2024), La-SP/SA maintained structural integrity across varying pH levels, highlighting the enhanced durability conferred by composite formation. In summary, La-SP/SA exhibited outstanding adsorption stability, supporting its potential as a reliable adsorbent for sustainable F^−^ removal in aqueous environments. It should be noted that this stability test reflects resistance to performance degradation under continuous exposure, rather than reusability, which would require systematic multi-cycle adsorption-desorption studies.

**FIGURE 5:**
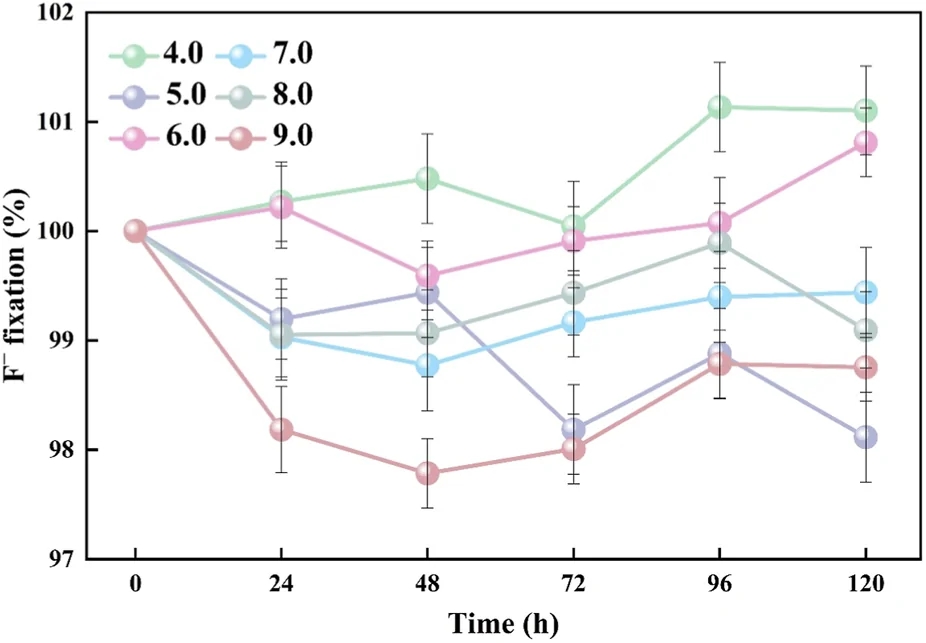
Adsorption stability test.

#### 3.4.2 fluoride removal tests in the anion surroundings

The interference of coexisting anions and natural organic matter on F^−^ removal was systematically investigated. As shown in Figures 6a–d, the presence of Cl^−^, HCO_3_^−^, NO_3_^−^ and SO_4_^2−^ showed minimal interference, with F^−^ removal efficiency remaining above 87.14%. Xu et al. (2026) also reported similar results. In contrast, PO_4_^3−^ exerted the most pronounced inhibitory effect (Figure 6e), with the capacity loss of La-SP/SA reaching 26.79% at 10 mg L^−1^. This strong competition was attributed to its ability to form inner-sphere complexes with the metal sites on La-SP/SA, thereby directly competing with F^−^ for active centers (Liu et al., 2024). The influence of humic acid (HA), as a representative natural organic substance, was examined at concentrations of 10–100 mg L^-1^. Figure 6f showed that the F^−^ removal efficiency decreased to 61.52% as the HA concentration increased to 100 mg L^−1^. This was attributed to the ability of La^3+^ to bind with HA via strong coordination bonds (Wang et al., 2024). Notably, the presence of HA did not significantly occupy the available adsorption sites. Overall, while PO_4_^3−^ and HA showed the strongest competitive effects under elevated concentrations, their interference at environmentally realistic levels in most groundwater sources is expected to be moderate (Wang et al., 2026; Wit et al., 2025). For applications in high-phosphate or high-organic matter waters, appropriate pre-treatment could be explored to enhance practical feasibility. Therefore, the material has potential applicability in real aquatic environments with complex matrices.

**FIGURE 6:**
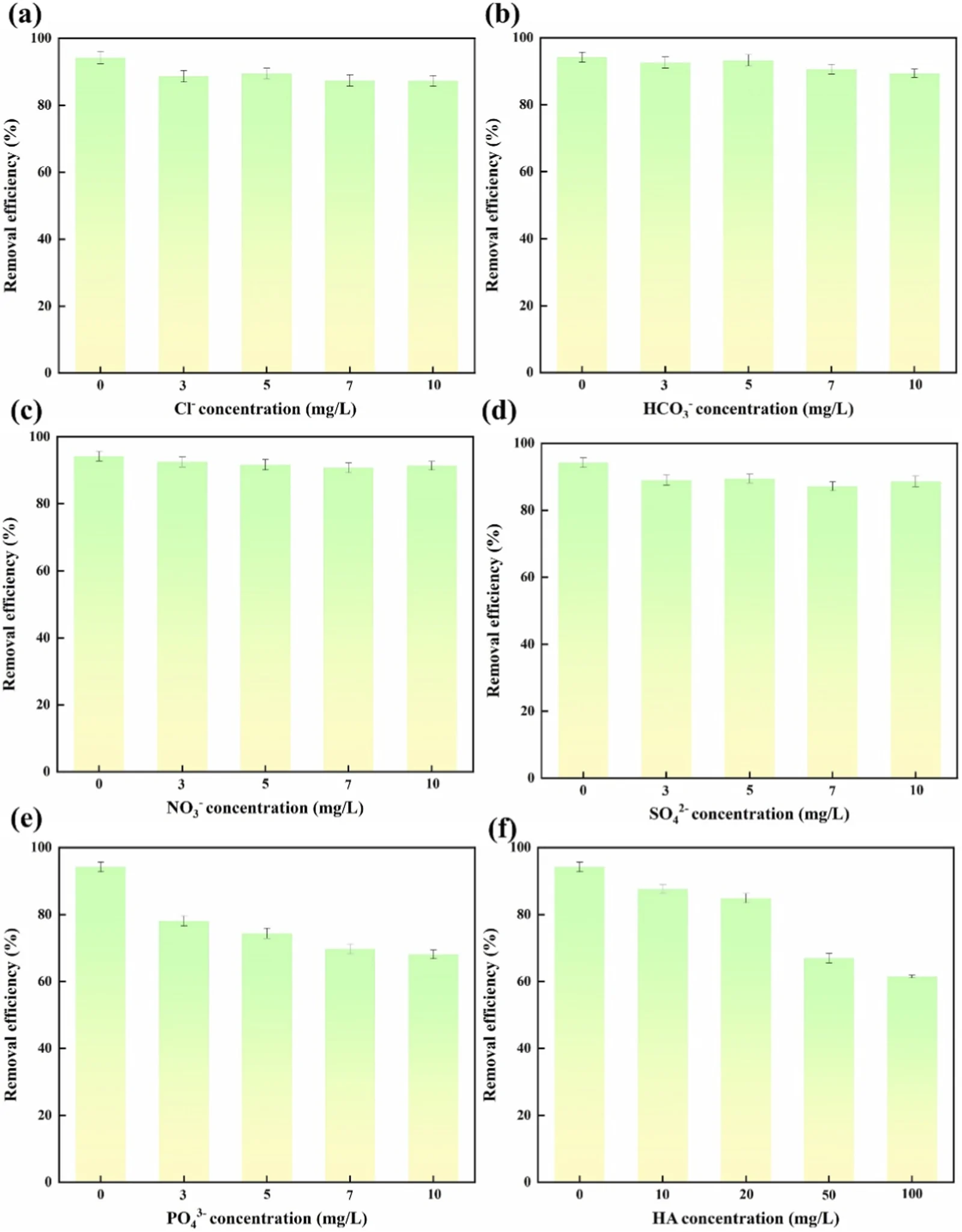
The F^−^ adsorption in the **(a)** Cl^−^, **(b)** HCO_3_^−^, **(c)** NO_3_^−^, **(d)** SO_4_^2−^, **(e)** PO_4_^3−^, and **(f)** HA surroundings.

### 3.5 Morphology of La-SP/SA

Before adsorption, the surface of La-SP/SA exhibited a rough morphology featuring pores and cracks of varying sizes (Figure 7a), which facilitated F^−^ adsorption (Zhou et al., 2024). EDS analysis confirmed the successful loading of La onto the hydrogel surface, and the detection of Ca and Si signals further indicated the successful incorporation of SP into the hydrogel matrix. After F^−^ adsorption, flake-like aggregates appeared on the hydrogel surface (Figure 7b). Concurrently, the concentrations of La^3+^ and Ca^2+^ in the solution decreased markedly in the presence of F^−^. Among them, the La^3+^ dropped from 42.7 mg L^−1^ (fluoride-free system) to 18.7 mg L^−1^, while Ca^2+^ showed a similar reduction (Figure 8). These decreases further comfirmed that the flake-like crystals were formed via chemical precipitation. EDS results demonstrated that F^−^ has been successfully immobilized by the hydrogel. Furthermore, the Cl^−^ content on the hydrogel surface after F^−^ adsorption decreased from 9.60% to 0.17% (Figures 7a,b), while the Cl^−^ concentration in the leachate increased from 28.49 to 34.72 mg L^−1^ (Figure 8). These phenomena indicated an ion exchange reaction between F^−^ and the free Cl^−^ present within La-SP/SA. It should be noted that the La^3+^ level observed (18.7 mg/L in the fluoride-containing system) warrants attention for practical applications, although no specific lanthanum limit currently exists in WHO, U.S. EPA, or Chinese drinking water standards. Further studies are needed to evaluate La^3+^ release under real groundwater matrices and to explore strategies for minimizing La leaching during long-term operation.

**FIGURE 7:**
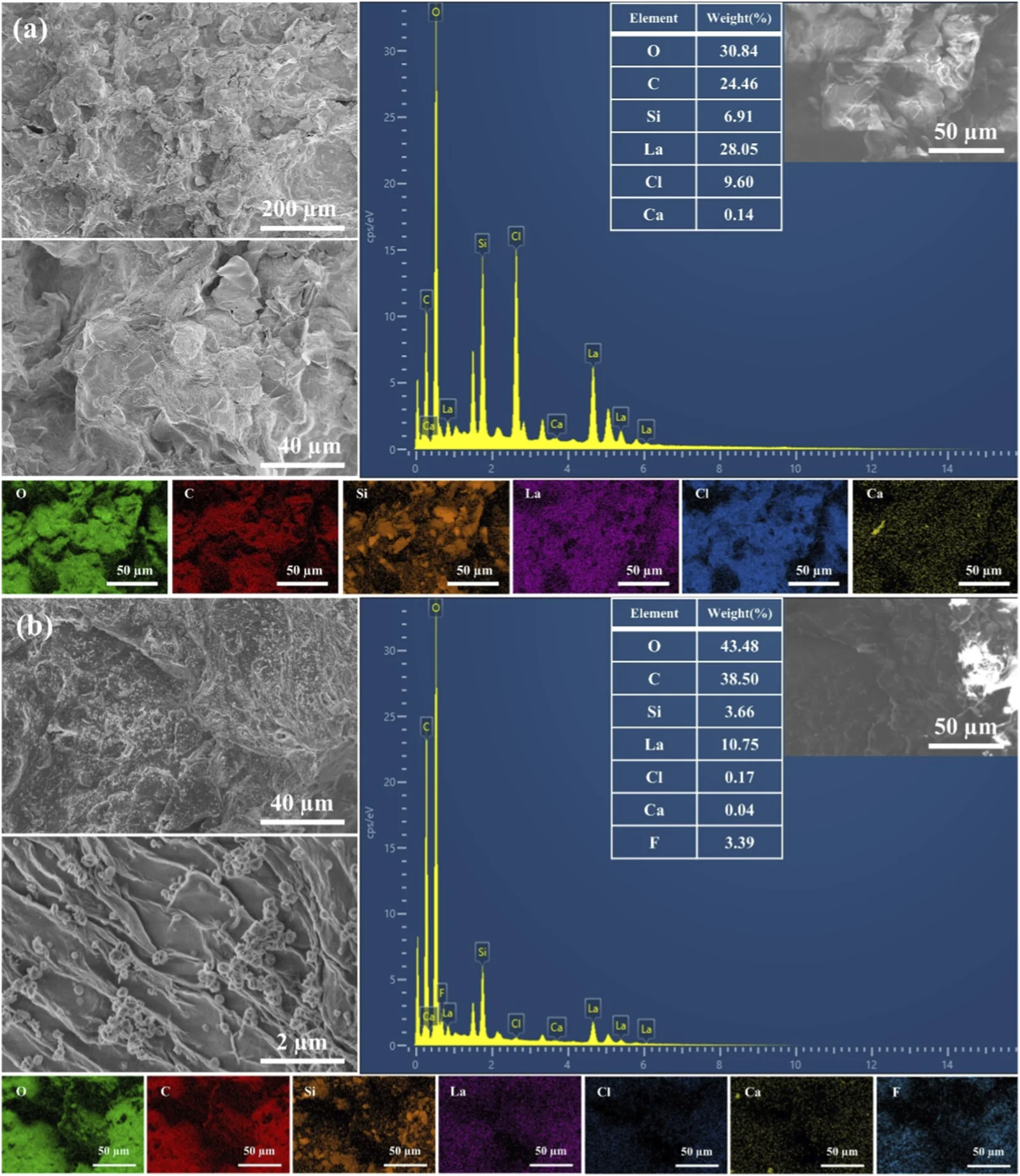
SEM-EDS images of La-SP/SA **(a)** before and **(b)** after F^−^ adsorption.

**FIGURE 8:**
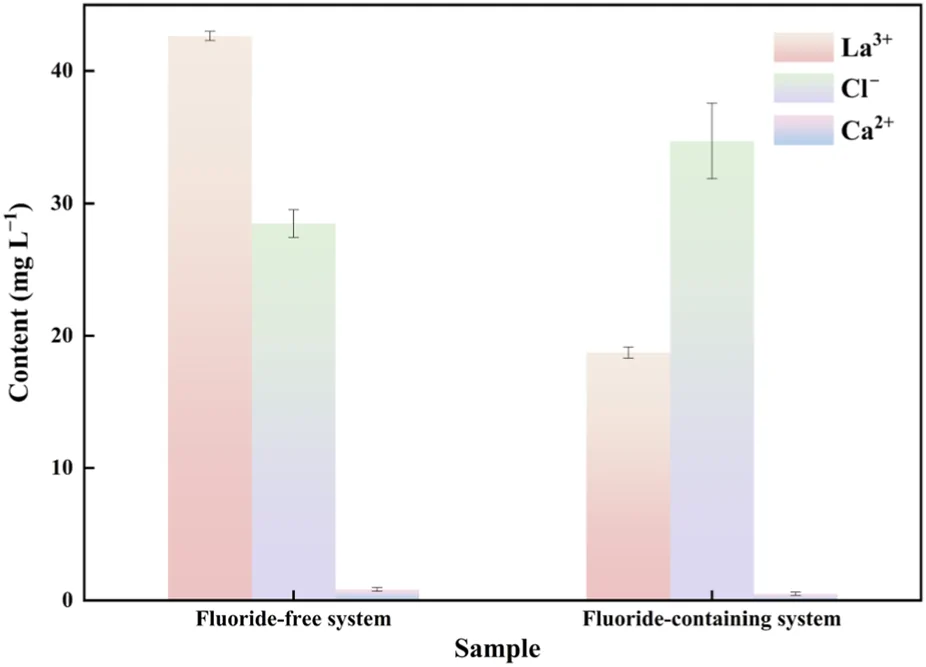
Contents of La^3+^, Ca^2+^ and Cl^−^ in the fluoride-free and fluoride-containing systems after reaction.

### 3.6 fluoride removal mechanism

#### 3.6.1 FTIR analysis

The FTIR spectra of La-SP/SA before and after F^−^ adsorption were presented in Figure 9a. Before adsorption, absorption peaks were observed at 3435, 1632, 1423, and 1033 cm^−1^, corresponding to O−H stretching vibration, −COO asymmetric stretching vibration, −COO symmetric stretching vibration, and C−O−C stretching vibration, respectively (Yu et al., 2021; Zhan et al., 2022). After adsorption, the O−H stretching peak shifted to 3437 cm^−1^, which can be attributed to inner-sphere complexation of F^−^ with hydroxylated metal sites (M−OH) (Xu et al., 2026). Moreover, the characteristic peaks of −COO and C−O−C appeared at 1417 and 1032 cm^−1^, respectively. These indicated that the surface functional groups of SA served as an important pathway for F^−^ adsorption onto La-SP/SA.

**FIGURE 9:**
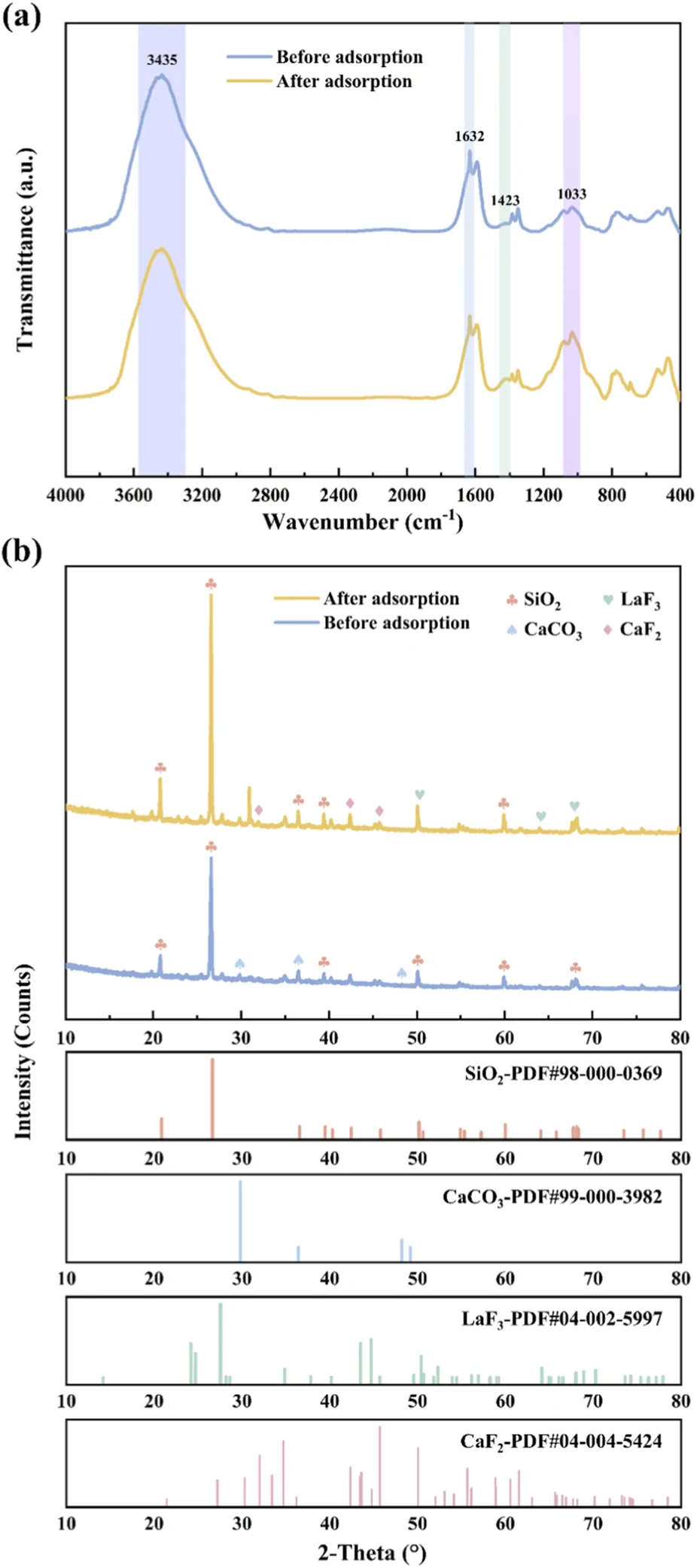
**(a)** FTIR and **(b)** XRD patterns before and after F^−^ adsorption.

#### 3.6.2 XRD analysis

The crystal structures of La-SP/SA before and after F^−^ adsorption were characterized by XRD (Figure 9b). The diffraction peaks at 20.9°, 26.6°, 39.5°, 50.1°, 60.0°, and 68.1° were assigned to SiO_2_ (PDF#98–000–0369), while those at 29.8°, 36.4°, and 48.2° corresponded to CaCO_3_ (PDF#99-000-3982). These results were consistent with the EDS analysis, confirming that SP was successfully loaded onto the composite hydrogel. After F^−^ adsorption, diffraction peaks appeared at 50.4° (302), 64.1° (214), and 68.0° (034), verifying the formation of LaF_3_ crystals (PDF#04–002–5997). Characteristic peaks observed at 32.0° (102), 42.3° (112), and 45.7° (211) corresponded to CaF_2_ (PDF#04–004–5424). The emergence of these new crystalline phases further confirmed the formation of fluoride-containing precipitates involving Ca and La. Notably, the detected CaF_2_ likely originated from two distinct pathways: (a) chemisorption between F^−^ and CaCO_3_ inherently present in SP (Li S. S. et al., 2025), and (b) chemical precipitation of F^−^ with Ca^2+^ released from the hydrogel matrix (Section 3.5).

#### 3.6.3 XPS analysis

As shown in Figure 10a, characteristic signals of La 3d, C 1s, and Si 2p were detected in the sample before adsorption, consistent with the EDS results, further confirming the successful preparation of the target material. After adsorption, a high-resolution F 1s spectrum emerged at 685.13 eV (Figure 10c), demonstrating that F^−^ was successfully immobilized (Liu F. et al., 2025). As shown in Figure 10b, the binding energy of the C−O−C bond shifted to 286.25 eV, whereas that of the O−C=O bond shifted to 288.38 eV (Fan et al., 2022). These changes corroborated the conclusion from FTIR analysis that the surface functional groups of SA participated in the F^−^ adsorption process. According to Figure 10d, the characteristic peaks of La 3d3/2 and La 3d5/2, located at 855.28/851.92 eV and 838.60/835.31 eV (Yu S.-H. et al., 2025), shifted to 853.82/850.73 eV and 840.52/837.10 eV. The significant changes demonstrated that F^−^ coordinated with La^3+^ on the hydrogel surface to form inner-sphere complexes (Zhou et al., 2024).

**FIGURE 10:**
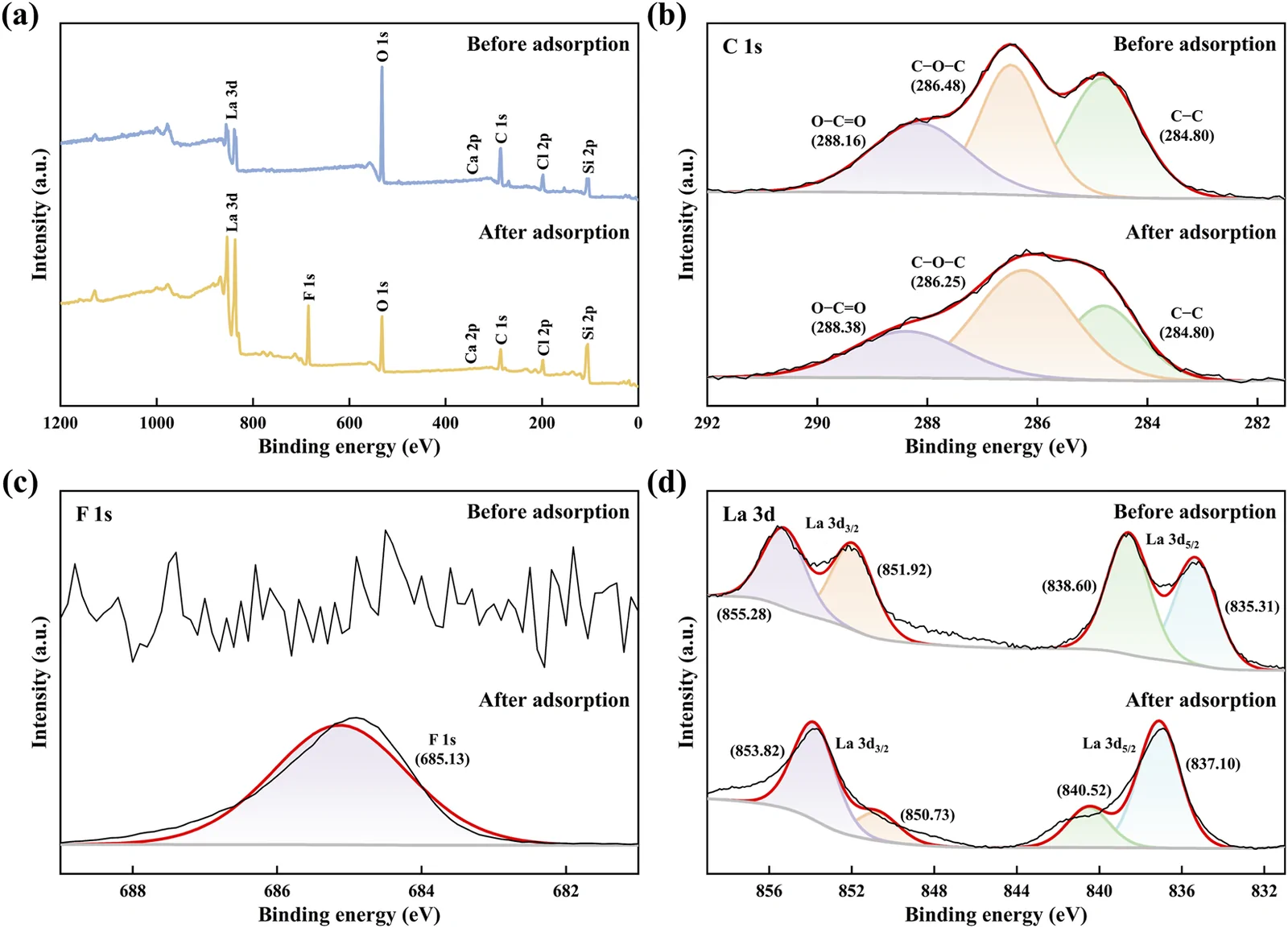
XPS spectrogram before and after F^−^ adsorption: **(a)** full-range scan spectra, **(b)** C 1s, **(c)** F 1s, and **(d)** La 3d.

#### 3.6.4 fluoride removal mechanism

Based on the above discussion, the following defluorination pathways were proposed for La-SP/SA (Figure 11). Four pathways are confirmed by direct characterization evidence: (a) Under acidic conditions (pH < 6.18), protonation of the hydrogel surface triggered electrostatic attraction of F^−^; (b) Free Cl^−^ within the hydrogel was replaced by adjacent F^−^; (c) Ligand exchange between F^−^ and M−OH produced more stable inner-sphere complexes; (d) The F^−^ and metal ions (La^3+^ and Ca^2+^) formed flaky microcrystalline precipitates on the hydrogel surface via chemical precipitation. Two additional pathways are proposed requiring further verification: (a) Chemisorption occurred between the F^−^ and CaCO_3_ originating from SP; (b) SA surface groups (C=O and C−O) participated in the F^−^ adsorption.

**FIGURE 11:**
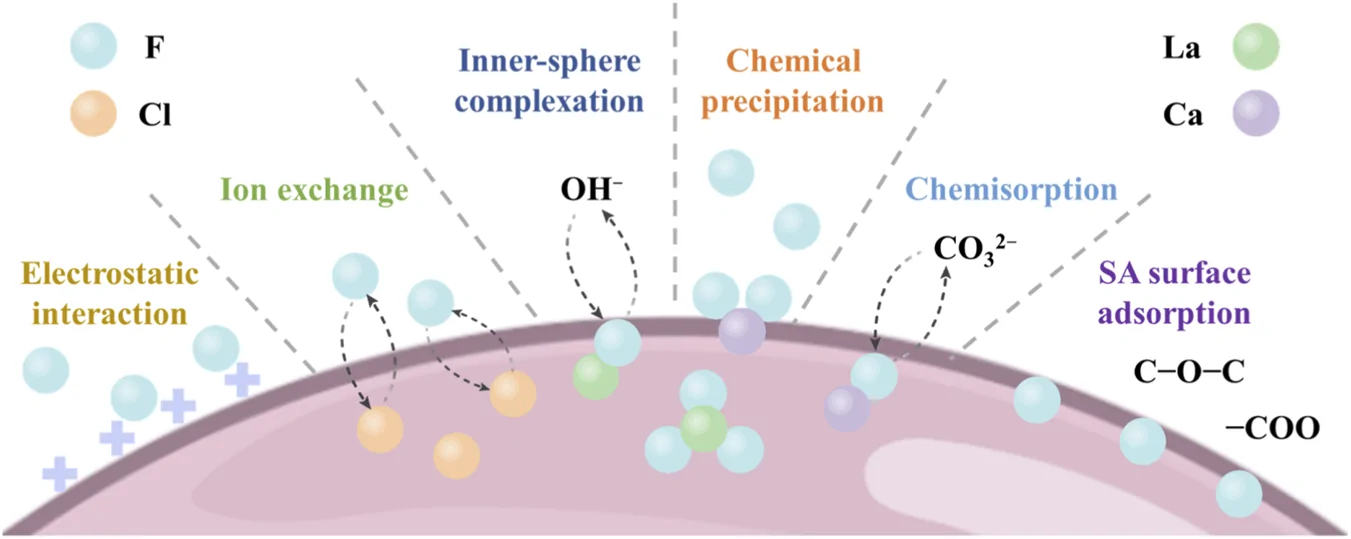
Proposed removal mechanisms of F^−^ by La-SP/SA.

## 4 Conclusion

In this study, La-SP/SA composite hydrogel was successfully developed for efficient defluorination. The synthesis parameters (SP dosage, La^3+^ content, and crosslinking time) and operational parameters (F^−^ concentration, La-SP/SA dosage, and initial pH) were optimized, yielding a maximum F^−^ removal efficiency of 94.33% (12.18 mg g^−1^) under the tested conditions. Kinetic analysis confirmed that the adsorption process was well described by the pseudo-second-order model, and chemisorption was the rate-controlling step. Characterization analyses revealed that electrostatic interaction, ion exchange, inner-sphere complexation, chemical precipitation, and chemisorption were the primary defluorination mechanisms. Notably, La-SP/SA exhibited strong resistance to interference from coexisting anions and humic acid, retaining over 98.01% of its initial adsorption capacity within 120 h. These results demonstrate the potential of the La-SP/SA composite hydrogel for the remediation of fluoride-contaminated groundwater, providing a valuable reference for the resource utilization of SP and the development of high-performance hydrogel adsorbents.

## Data Availability

The raw data supporting the conclusions of this article will be made available by the authors, without undue reservation.

## References

[B1] Aljohani M. M. (2025). Biopolymer-encapsulated Al/Tb-Layered double hydroxide hydrogel beads for efficient Cr(VI) removal: green synthesis, Box–Behnken optimization, and comprehensive adsorption mechanism analysis. J. Water Process Eng. 80, 109215. 10.1016/j.jwpe.2025.109215

[B2] Azzeddine T. Marrane S. E. Goudali O. El Kaim Billah R. Boudouma A. Chaouiki A. (2024). Simple and modified chitosan gel beads from a natural source as a bio-sorbent for water defluoridation: experimental and computational perspectives. Inorg. Chem. Commun. 167, 112752. 10.1016/j.inoche.2024.112752

[B3] Behera B. Sahu H. B. (2023). Coal mine waste characterization and defluoridation property. Heliyon 9, e13244. 10.1016/j.heliyon.2023.e13244 36798783 PMC9925952

[B4] Bi X. Wang Z. Wu Z. Li X. Xu Y. Feng G. (2026). Calcium-rich biochar/lanthanum alginate hydrogel beads for fluoride removal from groundwater. Int. J. Biol. Macromol. 365, 152325. 10.1016/j.ijbiomac.2026.152325 42070606

[B5] Chang P.-H. Yang Y. Wen Y. Ahmed M. M. M. Chang J.-C. Chang T.-Y. (2024). Efficient ethidium bromide removal using sodium alginate/graphene oxide composite beads: insights into adsorption mechanisms and performance. Chem. Eng. J. 500, 156379. 10.1016/j.cej.2024.156379

[B6] Dar F. A. Kurella S. (2024). Comprehensive batch studies on removal of fluoride from aqueous solution by acid and alkali-activated adsorbents prepared from Dal lake weeds: mechanism, kinetics and thermodynamics. Environ. Res. 259, 119493. 10.1016/j.envres.2024.119493 38944101

[B7] Dong S. Li Y. Zhu K. Wang C. Zhai S. (2025). Advances in structure designing and function tailoring strategy toward alginate-based hydrogels for efficient water remediation: a review. Int. J. Biol. Macromol. 304, 140801. 10.1016/j.ijbiomac.2025.140801 39924010

[B8] Fan X. Wang X. Cai Y. Xie H. Han S. Hao C. (2022). Functionalized cotton charcoal/chitosan biomass-based hydrogel for capturing Pb^2+^, Cu^2+^ and MB. J. Hazard. Mater. 423, 127191. 10.1016/j.jhazmat.2021.127191 34537654

[B9] Foroutan R. Tutunchi A. Foroughi M. Ramavandi B. (2025). Efficient fluoride removal from water and industrial wastewater using magnetic chitosan/β-cyclodextrin aerogel enhanced with biochar and MOF composites. Sep. Purif. Technol. 363, 132128. 10.1016/j.seppur.2025.132128

[B10] He J. Yang Y. Wu Z. Xie C. Zhang K. Kong L. (2020). Review of fluoride removal from water environment by adsorption. J. Environ. Chem. Eng. 8, 104516. 10.1016/j.jece.2020.104516

[B11] He D. Zhang Z. Zhang W. Zhang H. Liu J. (2024). Municipal sludge biochar skeletal sodium alginate beads for phosphate removal. Int. J. Biol. Macromol. 261, 129732. 10.1016/j.ijbiomac.2024.129732 38280708

[B12] Hussain Md.I. Rahman N. A. Dutta H. Dutta D. Dutta R. R. (2025). ZnO nanoparticles coated rice husk bio adsorbent for the removal of arsenic, phosphate and fluoride from contaminated water samples. Next Sustain. 5, 100129. 10.1016/j.nxsust.2025.100129

[B13] Huyen D. T. Phat L. N. Tien D. X. Thu D. P. G. Thoai D. Q. (2023). Bone-char from various food-waste: synthesis, characterization, and removal of fluoride in groundwater. Environ. Technol. and Innovation 32, 103342. 10.1016/j.eti.2023.103342

[B14] Jeyaseelan A. Viswanathan N. Kumar I. A. Ansar S. (2023). Effective defluoridation using lanthanum-organic frameworks encapsulated hydrotalcite based bio-hybrid beads. J. Solid State Chem. 328, 124301. 10.1016/j.jssc.2023.124301

[B15] Jian S. Liu J. Wu J. Yang W. Ran L. Hu J. (2025). Glutaraldehyde-crosslinked chitosan microspheres encapsulating La(OH)3 by electrospraying for efficient remediation of fluoride ions. Chem. Eng. J. 512, 162470. 10.1016/j.cej.2025.162470

[B16] Lacson C. F. Z. Lu M.-C. Huang Y.-H. (2021). Fluoride-containing water: a global perspective and a pursuit to sustainable water defluoridation management -An overview. J. Clean. Prod. 280, 124236. 10.1016/j.jclepro.2020.124236

[B17] Li B. Zhou X. Su N. Zhao Q. (2025). Facile preparation and exceptional surface adsorbability of Lanthanum(III)-crosslinked sodium alginate doped iron(III)-modified biochar biogel composite for fluoride. Surfaces Interfaces 72, 107174. 10.1016/j.surfin.2025.107174

[B18] Li S. S. Wang Y. H. Yang Z. P. Liu P. Ren Y. T. Li Y. (2025). Magnetic recyclable metal-organic framework sheets grafted with curcumin for highly efficient adsorption and sensitive detection of fluoride. Talanta 293, 128156. 10.1016/j.talanta.2025.128156 40239589

[B19] Linhardt P. (2026). Electrochemistry in remote places for assessing the corrosiveness in natural waters. Electrochimica Acta 554, 148293. 10.1016/j.electacta.2026.148293

[B20] Liu R. Song J. Zhao J. Wang Z. Xu J. Yang W. (2024). Novel MOF(Zr)–on-MOF(Ce/La) adsorbent for efficient fluoride and phosphate removal. Chem. Eng. J. 497, 154780. 10.1016/j.cej.2024.154780

[B21] Liu F. Wang Q. Li Y. Zhou Z. Wang N. Wang T. (2025). 3D crosslinked chitosan for fluoride remediation in industrial wastewater: from structure to performance enhancement. Sep. Purif. Technol. 360, 131070. 10.1016/j.seppur.2024.131070

[B22] Liu S. Wang J. Ren J. Zhang M. Mao H. (2025). Environmentally benign tannin-modified chitosan aerogel decorated with Zr(IV) enabled high-performance adsorption of fluoride. Chem. Eng. J. 519, 165351. 10.1016/j.cej.2025.165351

[B23] Njaramba L. K. Kim S. Kim Y. Cha B. Kim N. Yoon Y. (2022). Remarkable adsorption for hazardous organic and inorganic contaminants by multifunctional amorphous core–shell structures of metal–organic framework-alginate composites. Chem. Eng. J. 431, 133415. 10.1016/j.cej.2021.133415

[B24] Oke E. A. Oluyinka O. A. Afolabi S. D. Ibe K. K. Raheem S. A. (2023). Latest insights on technologies for halides and halogenated compounds extraction/abatement from water and wastewater: challenges and future perspectives. J. Water Process Eng. 53, 103724. 10.1016/j.jwpe.2023.103724

[B25] Ratthika S. A. Ramkumar K. Elanchezhiyan S.Sd. Kumar R. Meenakshi S. (2025). Synthesis and characterization of lanthanum-assisted aluminium metal-organic frameworks for the removal of fluoride ions from an aqueous environment. J. Environ. Chem. Eng. 13, 118181. 10.1016/j.jece.2025.118181

[B26] Rosendo-González V. Gutiérrez-Segura E. Solache-Rios M. Amaya-Chavez A. (2024). Removal of fluoride ions from aqueous solutions on unmodified and iron-modified hydrogels. J. Polym. Res. 31, 133. 10.1007/s10965-024-03954-0

[B27] Shen D. Song Y. Chen X. Zhou Y. Li H. Pan J. (2022). Fabricating ultrafine zirconium oxide based composite sorbents in “soft confined space” for efficiently removing fluoride from environmental water. Chem. Eng. J. 444, 136199. 10.1016/j.cej.2022.136199

[B28] Thakur R. Patial B. (2026). Neglected parathyroids: fluoride’s trigger for skeletal fluorosis and endocrine domino effect. J. Trace Elem. Med. Biol. 96, 127916. 10.1016/j.jtemb.2026.127916 42335482

[B29] Wang Z. Wu Z. (2025). Insights into fluorine stabilization by biological approaches: mechanisms, toxicity regulatory, application feasibility, and new challenges. Bioresour. Technol. 434, 132854. 10.1016/j.biortech.2025.132854 40543822

[B30] Wang B. Hu X. Zhou D. Zhang H. Chen R. Guo W. (2021). Highly selective and sustainable clean-up of phosphate from aqueous phase by eco-friendly lanthanum cross-linked polyvinyl alcohol/alginate/palygorskite composite hydrogel beads. J. Clean. Prod. 298, 126878. 10.1016/j.jclepro.2021.126878

[B31] Wang Z. Su J. Zhao T. Li J. Zhang L. (2024). Enhanced removal of fluoride from groundwater using biosynthetic hydroxyapatite modified by bimetallic (La–Fe or La–Al) hydroxides. J. Clean. Prod. 436, 140649. 10.1016/j.jclepro.2024.140649

[B32] Wang C. Jiao J. Wang X. Jin G. Huang P. Jiang K. (2026). Contrasting short-term changes in nutrients and dissolved organic matter in groundwater and surface water on a coastal island following super typhoon yagi. Mar. Environ. Res. 221, 108336. 10.1016/j.marenvres.2026.108336 42580298

[B33] Wit M. R. J. van Egmond J. L. Kruijssen T. P. Bense V. F. van Breukelen B. M. (2025). Hydrogeochemical signatures and human impact: a comprehensive analysis of groundwater quality on the semi-arid island of Curaçao. J. Hydrology Regional Stud. 60, 102555. 10.1016/j.ejrh.2025.102555

[B34] Xu Y. Lun Z. Wang H. Hu W. Zhao C. Zhou X. (2025). SO_2_ ad-/desorption performance on shale matrix: new insights into SO mitigation via geologic storage in unconventional natural gas reservoir. Chem. Eng. J. 507, 160422. 10.1016/j.cej.2025.160422

[B35] Xu Y. Wang Z. Wu Z. Li X. Bi X. Feng G. (2026). Lanthanum-calcium modified coal gangue-alginate composite hydrogel beads for efficient fluoride removal from groundwater. Inorg. Chem. Commun. 185, 116101. 10.1016/j.inoche.2025.116101

[B36] Yan J. Li K. (2021). A magnetically recyclable polyampholyte hydrogel adsorbent functionalized with β-cyclodextrin and graphene oxide for cationic/anionic dyes and heavy metal ion wastewater remediation. Sep. Purif. Technol. 277, 119469. 10.1016/j.seppur.2021.119469

[B37] Yu T. Chen Y. Zhang Y. Tan X. Xie T. Shao B. (2021). Novel reusable sulfate-type zirconium alginate ion-exchanger for fluoride removal. Chin. Chem. Lett. 32, 3410–3415. 10.1016/j.cclet.2021.04.057

[B38] Yu M. Gan Z. Liao T. Tang A. Yang H. (2025). Interfacial hydration of zeolite-confined MgO for enhanced fluoride removal from water. Chem. Eng. J. 513, 163053. 10.1016/j.cej.2025.163053

[B39] Yu S.-H. Feng X.-Y. Li H. Chong H.-B. Hua L. (2025). Magnetic lanthanum-modified ball-milled steel slag encapsulated alginate composite hydrogel beads for low-level phosphate removal. Int. J. Biol. Macromol. 321, 146308. 10.1016/j.ijbiomac.2025.146308 40716547

[B40] Yusuff A. S. Popoola L. T. Ibrahim I. S. (2025). Adsorptive removal of anthraquinone dye from wastewater using silica-nitrogen reformed eucalyptus bark biochar: parametric optimization, isotherm and kinetic studies. J. Taiwan Inst. Chem. Eng. 166, 105503. 10.1016/j.jtice.2024.105503

[B41] Zeng Z. Li Q. Yan J. Huang L. Arulmani S. R. B. Zhang H. (2023). The model and mechanism of adsorptive technologies for wastewater containing fluoride: a review. Chemosphere 340, 139808. 10.1016/j.chemosphere.2023.139808 37591373

[B42] Zhan J. Sun H. Xie M. Han J. Chen L. Zhao Y. (2022). Hyperbranched polyamidoamine-chitosan polyelectrolyte gels crosslinking by polyacrylic acid and alginate for removal of anionic dyes. Int. J. Biol. Macromol. 222, 3024–3033. 10.1016/j.ijbiomac.2022.10.077 36244541

[B43] Zhang Q. Zhou Y. Yao Q. Zhang F. Chen W. Liu Y. (2022). Comparison of fluorine removal performance and mechanism of spheroidal magnesium oxide before and after lanthanum modification. Environ. Sci. Pollut. Res. 29, 80477–80490. 10.1007/s11356-022-21177-y 35717549

[B44] Zhang Y. Xie Q. Fu C. Qiu Z. Zhuang Z. Wang T. (2025). Lanthanum-based hydrogels for simultaneous removal of trace phosphorus and algae. Chem. Eng. J. 519, 164822. 10.1016/j.cej.2025.164822

[B45] Zhao X. Zhang Y. Xue N. Hu P. (2025). Research on the mechanism of mechanochemical activation for enhanced leaching of vanadium-bearing shale: activation kinetics and fluorine adsorption. RSC Adv. 15, 25233–25249. 10.1039/D5RA03987H 40673248 PMC12265454

[B46] Zhou X. Li B. Zhao Q. (2024). Effective removal and adsorption mechanism of fluoride from water by biochar-based Ce(III)-La(III)-crosslinked sodium alginate hybrid hydrogel. Int. J. Biol. Macromol. 272, 132925. 10.1016/j.ijbiomac.2024.132925 38844281

