# Supplementary material for: Lanthanum-modified shale powder/alginate composite hydrogel beads for fluoride-contaminated groundwater treatment
Source: Front Chem. 2026 Sep 23;14:1962172. doi: 10.3389/fchem.2026.1962172 (PMC13642737; doi:10.3389/fchem.2026.1962172)
Supplement: Supplementary file 1 [file Table1.docx]

**Supplementary Material**

**Lanthanum-modified** **shale powder/alginate composite hydrogel beads for fluoride-contaminated** **groundwater** **treatment**

Sitong Liu ^a#^, Shaoxiong Li ^b#^, Xu Bi ^b^, Guangying Feng ^b^, Zhao Wang ^b*^, Qiusheng Yuan ^b*^, Yue Xu ^b^, Ningyi Li ^b^

^a^ Academic Affairs Office, Kunming Railway Vocational and Technical College, Kunming 650208, Yunnan, China.

^b^ Faculty of Environmental Science & Engineering, Kunming University of Science & Technology, Kunming 650500, Yunnan, China.

^#^ The authors contributed equally to this work.

**Corresponding author**

**Zhao Wang**

Faculty of Environmental Science and Engineering,

Kunming University of Science and Technology, Kunming 650500, China.

E-mail address: 20240157@kust.edu.cn

**Qiusheng Yuan**

Faculty of Environmental Science and Engineering,

Kunming University of Science and Technology, Kunming 650500, China.

E-mail address: [hhuenvyuan@kust.edu.cn](mailto:20250180@kust.edu.cn)

Table S1. Kinetic parameters at different SP dosage.

| Kinetic Model | Parameters | SP dosage (%) | | | | |
| --- | --- | --- | --- | --- | --- | --- |
|  |  | **0.0** | **1.0** | **2.0** | **4.0** | **6.0** |
| Pseudo-first-order | **k_1_ (h^−1^)** | 0.4289 | 0.5223 | 0.5385 | 0.5623 | 0.5276 |
|  | **R^2^** | 0.6097 | 0.7895 | 0.7884 | 0.7596 | 0.7242 |
|  | **q_e_, _exp_ (mg g^−1^)** | 9.00 | 9.13 | 9.15 | 9.23 | 9.14 |
|  | **q_e_, _cal_ (mg g^−1^)** | 2.03 | 2.71 | 2.57 | 2.18 | 2.15 |
| Pseudo-second-order | **k_2_ (g mg^−1^ h^−1^)** | 0.9646 | 0.8423 | 0.8939 | 1.0312 | 1.0137 |
|  | **R^2^** | 0.9998 | 0.9998 | 0.9999 | 0.9999 | 0.9999 |
|  | **q_e_, _cal_ (mg g^−1^)** | 9.05 | 9.24 | 9.25 | 9.31 | 9.21 |
| Intra-particle diffusion | **k_1_ (mg g^-1^ h^0.5^)** | 0.9937 | 0.7379 | 0.8390 | 0.7177 | 0.9138 |
|  | **R_1_^2^** | 0.8078 | 0.7827 | 0.7279 | 0.9734 | 0.7550 |
|  | **k_2_ (mg g^-1^ h^0.5^)** | 0.2639 | 0.5525 | 0.5079 | 0.4324 | 0.2894 |
|  | **R_2_^2^** | 0.7020 | 0.9763 | 0.9702 | 0.8852 | 0.8157 |
|  | **k_3_ (mg g^-1^ h^0.5^)** | 0.2856 | 0.2673 | 0.2189 | 0.1324 | 0.2211 |
|  | **R_3_^2^** | 0.9974 | 0.8385 | 0.9412 | 0.9989 | 0.8582 |

Table S2. Kinetic parameters at different La^3+^ content.

| Kinetic Model | Parameters | La^3+^ content (mol L^−1^) | | | | |
| --- | --- | --- | --- | --- | --- | --- |
|  |  | **0.1** | **0.2** | **0.5** | **1.0** | **2.0** |
| Pseudo-first-order | **k_1_ (h^−1^)** | 0.6108 | 0.5790 | 0.5453 | 0.5136 | 0.5608 |
|  | **R^2^** | 0.7576 | 0.6609 | 0.6454 | 0.6184 | 0.6805 |
|  | **q_e_, _exp_ (mg g^−1^)** | 8.88 | 9.07 | 9.07 | 9.06 | 9.02 |
|  | **q_e_, _cal_ (mg g^−1^)** | 1.85 | 1.43 | 1.51 | 1.53 | 1.64 |
| Pseudo-second-order | **k_2_ (g mg^−1^ h^−1^)** | 1.2079 | 1.4106 | 1.3192 | 1.2704 | 1.2566 |
|  | **R^2^** | 0.9999 | 0.9999 | 0.9999 | 0.9999 | 0.9999 |
|  | **q_e_, _cal_ (mg g^−1^)** | 8.94 | 9.11 | 9.11 | 9.10 | 9.06 |
| Intra-particle diffusion | **k_1_ (mg g^-1^ h^0.5^)** | 0.7283 | 0.4865 | 0.5787 | 0.5397 | 0.7273 |
|  | **R_1_^2^** | 0.8873 | 0.7443 | 0.6898 | 0.6708 | 0.5279 |
|  | **k_2_ (mg g^-1^ h^0.5^)** | 0.2939 | 0.2085 | 0.2204 | 0.2552 | 0.2670 |
|  | **R_2_^2^** | 0.9403 | 0.7032 | 0.7838 | 0.8040 | 0.9099 |
|  | **k_3_ (mg g^-1^ h^0.5^)** | 0.0935 | 0.0949 | 0.1152 | 0.1247 | 0.1059 |
|  | **R_3_^2^** | 0.9777 | 0.9687 | 0.9915 | 0.9895 | 0.9997 |

Table S3. Kinetic parameters under different cross-linking time.

| Kinetic Model | Parameters | Cross-linking time (h) | | | | |
| --- | --- | --- | --- | --- | --- | --- |
|  |  | **8** | **12** | **24** | **36** | **48** |
| Pseudo-first-order | **k_1_ (h^−1^)** | 0.5679 | 0.5003 | 0.4859 | 0.4705 | 0.4948 |
|  | **R^2^** | 0.6903 | 0.5966 | 0.5909 | 0.5854 | 0.6381 |
|  | **q_e_, _exp_ (mg g^−1^)** | 9.22 | 9.40 | 9.34 | 9.33 | 9.30 |
|  | **q_e_, _cal_ (mg g^−1^)** | 1.70 | 1.54 | 1.63 | 1.78 | 1.92 |
| Pseudo-second-order | **k_2_ (g mg^−1^ h^−1^)** | 1.2487 | 1.2600 | 1.1988 | 1.1133 | 1.0799 |
|  | **R^2^** | 0.9999 | 0.9999 | 0.9999 | 0.9999 | 0.9999 |
|  | **q_e_, _cal_ (mg g^−1^)** | 9.27 | 9.41 | 9.37 | 9.37 | 9.35 |
| Intra-particle diffusion | **k_1_ (mg g^-1^ h^0.5^)** | 0.7941 | 0.6790 | 0.7713 | 1.1652 | 1.1562 |
|  | **R_1_^2^** | 0.8882 | 0.9253 | 0.8686 | 0.6892 | 0.8140 |
|  | **k_2_ (mg g^-1^ h^0.5^)** | 0.2226 | 0.1829 | 0.2157 | 0.2194 | 0.2531 |
|  | **R_2_^2^** | 0.9978 | 0.9991 | 0.7650 | 0.9259 | 0.7980 |
|  | **k_3_ (mg g^-1^ h^0.5^)** | 0.1011 | 0.1331 | 0.1525 | 0.1706 | 0.1665 |
|  | **R_3_^2^** | 0.9976 | 0.9689 | 0.9375 | 0.8511 | 0.9280 |

Table S4. Kinetic parameters at different F⁻ concentration.

| Kinetic Model | Parameters | F⁻ concentration (mg L^−1^) | | | | |
| --- | --- | --- | --- | --- | --- | --- |
|  |  | **3.0** | **5.0** | **7.0** | **9.0** | **15** |
| Pseudo-first-order | **k_1_ (h^−1^)** | 0.4854 | 0.3986 | 0.3483 | 0.3760 | 0.4577 |
|  | **R^2^** | 0.8068 | 0.5094 | 0.2973 | 0.3361 | 0.4970 |
|  | **q_e_, _exp_ (mg g^−1^)** | 5.28 | 9.40 | 13.20 | 17.21 | 25.91 |
|  | **q_e_, _cal_ (mg g^−1^)** | 2.10 | 1.77 | 1.57 | 1.99 | 3.62 |
| Pseudo-second-order | **k_2_ (g mg^−1^ h^−1^)** | 0.8499 | 1.0357 | 1.1261 | 1.0219 | 0.8099 |
|  | **R^2^** | 0.9999 | 0.9998 | 0.9998 | 0.9999 | 0.9999 |
|  | **q_e_, _cal_ (mg g^−1^)** | 5.40 | 9.43 | 13.19 | 17.22 | 25.97 |
| Intra-particle diffusion | **k_1_ (mg g^-1^ h^0.5^)** | 1.6513 | 0.7305 | 0.3449 | 0.4106 | 1.4006 |
|  | **R_1_^2^** | 0.9005 | 0.6525 | 0.9397 | 0.6768 | 0.5070 |
|  | **k_2_ (mg g^-1^ h^0.5^)** | 0.3869 | 0.1818 | 0.1315 | 0.1926 | 0.4549 |
|  | **R_2_^2^** | 0.7278 | 0.5729 | 0.9994 | 0.9682 | 0.7897 |
|  | **k_3_ (mg g^-1^ h^0.5^)** | 0.1851 | 0.3096 | 0.3080 | 0.3595 | 0.4265 |
|  | **R_3_^2^** | 0.9621 | 0.9998 | 0.7963 | 0.9320 | 0.9817 |

Table S5. Kinetic parameters under different La-SP/SA dosage.

| Kinetic Model | Parameters | La-SP/SA dosage (g L^−1^) | | | | |
| --- | --- | --- | --- | --- | --- | --- |
|  |  | **0.1** | **0.3** | **0.5** | **0.7** | **1.0** |
| Pseudo-first-order | **k_1_ (h^−1^)** | 0.2870 | 0.5245 | 0.7338 | 0.6520 | 0.7105 |
|  | **R^2^** | 0.7038 | 0.6386 | 0.6679 | 0.6124 | 0.6759 |
|  | **q_e_, _exp_ (mg g^−1^)** | 30.24 | 23.64 | 16.86 | 12.10 | 8.39 |
|  | **q_e_, _cal_ (mg g^−1^)** | 16.06 | 4.28 | 1.70 | 1.24 | 0.96 |
| Pseudo-second-order | **k_2_ (g mg^−1^ h^−1^)** | 0.2151 | 0.7515 | 1.5760 | 1.7083 | 2.0331 |
|  | **R^2^** | 0.9795 | 0.9999 | 1.0000 | 0.9999 | 0.9999 |
|  | **q_e_, _cal_ (mg g^−1^)** | 31.08 | 23.75 | 16.90 | 12.13 | 8.41 |
| Intra-particle diffusion | **k_1_ (mg g^-1^ h^0.5^)** | 3.3192 | 1.6379 | 0.5447 | 0.1853 | 0.2184 |
|  | **R_1_^2^** | 0.8329 | 0.7134 | 0.4138 | 0.7522 | 0.6691 |
|  | **k_2_ (mg g^-1^ h^0.5^)** | 1.4432 | 0.3555 | 0.2267 | 0.1403 | 0.0951 |
|  | **R_2_^2^** | 0.1107 | 0.7091 | 0.6491 | 0.5541 | 0.8598 |
|  | **k_3_ (mg g^-1^ h^0.5^)** | 6.5326 | 0.5291 | 0.0470 | 0.0843 | 0.0498 |
|  | **R_3_^2^** | 0.7950 | 0.7761 | 0.9635 | 0.6977 | 0.6390 |

Table S6. Kinetic parameters at different initial pH.

| Kinetic Model | Parameters | Initial value | | | | |
| --- | --- | --- | --- | --- | --- | --- |
|  |  | **4.0** | **5.0** | **6.0** | **7.0** | **9.0** |
| Pseudo-first-order | **k_1_ (h^−1^)** | 0.7689 | 0.6581 | 0.7126 | 0.6868 | 0.6140 |
|  | **R^2^** | 0.7269 | 0.6422 | 0.7110 | 0.7358 | 0.6531 |
|  | **q_e_, _exp_ (mg g^−1^)** | 12.20 | 12.18 | 12.05 | 12.15 | 12.00 |
|  | **q_e_, _cal_ (mg g^−1^)** | 1.64 | 1.55 | 1.51 | 1.96 | 1.61 |
| Pseudo-second-order | **k_2_ (g mg^−1^ h^−1^)** | 1.5815 | 1.4823 | 1.5947 | 1.3071 | 1.3404 |
|  | **R^2^** | 0.9999 | 0.9999 | 1.0000 | 0.9999 | 0.9999 |
|  | **q_e_, _cal_ (mg g^−1^)** | 12.24 | 12.22 | 12.09 | 12.20 | 12.04 |
| Intra-particle diffusion | **k_1_ (mg g^-1^ h^0.5^)** | 0.3150 | 0.4175 | 0.4076 | 0.4147 | 0.4924 |
|  | **R_1_^2^** | 0.9580 | 0.7383 | 0.9973 | 0.9752 | 0.9943 |
|  | **k_2_ (mg g^-1^ h^0.5^)** | 0.1278 | 0.0892 | 0.1653 | 0.2334 | 0.1915 |
|  | **R_2_^2^** | 0.8010 | 0.7681 | 0.9708 | 0.9586 | 0.9998 |
|  | **k_3_ (mg g^-1^ h^0.5^)** | 0.0736 | 0.1296 | 0.0520 | 0.1087 | 0.0762 |
|  | **R_3_^2^** | 0.5361 | 0.5682 | 0.8640 | 0.6430 | 0.9997 |
